# Pathogen change of avian influenza virus in the live poultry market before and after vaccination of poultry in southern China

**DOI:** 10.1186/s12985-021-01683-0

**Published:** 2021-10-29

**Authors:** Jin Guo, Wentao Song, Xiansheng Ni, Wei Liu, Jingwen Wu, Wen Xia, Xianfeng Zhou, Wei Wang, Fenglan He, Xi Wang, Guoyin Fan, Kun Zhou, Haiying Chen, Shengen Chen

**Affiliations:** 1 The Collaboration Unit for Field Epidemiology of State Key Laboratory of Infectious Disease Prevention and Control, Jiangxi Provincial Key Laboratory of Animal-Origin and Vector-Borne Diseases, Nanchang Center for Disease Control and Prevention, Nanchang, 330038 People’s Republic of China; 2 School of Public Health, Jiangxi Provincial Key Laboratory of Preventive Medicine, Nanchang University, Nanchang, 330006 People’s Republic of China

**Keywords:** Avian influenza, Vaccine, Live poultry market, Subtype, Environment

## Abstract

**Background:**

The fifth wave of H7N9 avian influenza virus caused a large number of human infections and a large number of poultry deaths in China. Since September 2017, mainland China has begun to vaccinate poultry with H5 + H7 avian influenza vaccine. We investigated the avian influenza virus infections in different types of live poultry markets and samples before and after genotype H5 + H7 vaccination in Nanchang, and analyzed the changes of the HA subtypes of AIVs.

**Methods:**

From 2016 to 2019, we monitored different live poultry markets and collected specimens, using real-time reverse transcription polymerase chain reaction (RT-PCR) technology to detect the nucleic acid of type A avian influenza virus in the samples. The H5, H7 and H9 subtypes of influenza viruses were further classified for the positive results. The χ^2^ test was used to compare the differences in the separation rates of different avian influenza subtypes.

**Results:**

We analyzed 5,196 samples collected before and after vaccination and found that the infection rate of AIV in wholesale market (21.73%) was lower than that in retail market (24.74%) (P < 0.05). Among all the samples, the positive rate of sewage samples (33.90%) was the highest (P < 0.001). After vaccination, the positive rate of H5 and H7 subtypes decreased, and the positive rate of H9 subtype and untypable HA type increased significantly (P < 0.001). The positive rates of H9 subtype in different types of LPMs and different types of samples increased significantly (P < 0.01), and the positive rates of untypable HA type increased significantly in all environmental samples (P < 0.05).

**Conclusions:**

Since vaccination, the positive rates of H5 and H7 subtypes have decreased, but the positive rates of H9 subtypes have increased to varying degrees in different testing locations and all samples. This results show that the government should establish more complete measures to achieve long-term control of the avian influenza virus.

## Introduction

Influenza A virus is a single-stranded negative-sense RNA virus, which consists of eight gene fragments [1]. So far, 18 different hemagglutinins (HA, H1–H18) and 11 different neuraminidase (NA, N1–N11) have been found, forming a huge pool of influenza viruses [2]. Since 1959, highly pathogenic H5 and H7 subtype avian influenza viruses(AIVs) carrying different NA subtypes have caused a large number of disease outbreaks in poultry and wild birds all over the world [3]. In 1997, 18 people in Hong Kong were infected with the H5N1 virus and 6 people died. This shows for the first time that influenza viruses can cross the species barrier and pose a huge threat to humans [4]. In recent decades, human infections have been caused by various subtypes of AIVs, including H10N8, H5N6 and H9N2, most of which were reported for the first time in China. In 2013, H7N9 influenza virus first appeared in Shanghai and Anhui of China, and then quickly spread to other provinces of China, which brought great losses to China's poultry industry and caused serious human infection [5]. It is considered to be one of the viruses most likely to cause a human influenza pandemic [6]. Since 2013, there have been five outbreaks of H7N9 in China [7]. The number of reported cases in the fifth wave (766 out of 1567) accounted for almost half of the total number of human cases [8]. This has raised concerns about the next wave of epidemics. In order to cope with the fifth outbreak of highly pathogenic H7N9 AIV, millions of infected poultry were slaughtered [9]. As part of the control strategy, the Ministry of Agriculture and Rural Affairs designated eight vaccine companies in China to produce an inactivated H5 + H7 vaccine based on the H5 subtype avian influenza vaccine to control the spread of avian influenza, the application of which was initiated in chickens in September 2017, with an inoculation rate of over 67.3% [8, 10].

South China has always been regarded as the hypothetical center for the emergence of pandemic influenza viruses in the world [11]. In the first few waves of the H7N9 avian flu outbreak, most human infections came from eastern and southern China [7, 12]. Nanchang City in Jiangxi Province is located in the south of China, where Poyang Lake is located, and it is also very close to the Yangtze River Delta and Dongting Lake. Thousands of migratory birds that migrate long distances every year stop to breed and drink in Poyang Lake and Dongting Lake, where the AIV is very active. Since 2013, there have been cases of H7N9, H10N8 and other cases in Nanchang City. Epidemiological investigations show that most of the cases have had a history of poultry contact or were exposed to live poultry markets (LPMs) before the onset of illness [13, 14]. According to previous research [15, 16], live poultry contact and exposure history in the LPM are the potential sources and primary risk factors for human infection with AIV Therefore, this study analyzed the changes of AIVs in the LPM before and after H5 + H7 avian influenza vaccination in Nanchang City, South China. To understand the changes of the AIV, provide a basis for further prevention and control measures.

## Methods

### Object

Since September 2017, the Ministry of Agriculture and Rural Affairs has been vaccinating chickens, ducks, geese and other live poultry raised at live poultry farms in Nanchang with the H5 + H7 AIV vaccine. In general, farmed live birds receive their first dose of vaccine at 10 days of age, a second dose at one month of age, and a third dose at three months of age. However, fast-growing live birds are typically given only two doses of the vaccine before being shipped to live poultry markets. In this study, before vaccination in 2016–2017 and after vaccination in 2018–2019, four live poultry markets were selected as fixed monitoring points in four different counties of Nanchang City, including three live poultry retail markets and one live poultry wholesale market. Select as many stalls as possible in each live poultry market for sampling. A total of 5196 samples of throat swabs (2576), cloacal swabs (110), feces (1014), surface smears of cages (1027) and sewage (469) were collected.

### Methods

The samples were immediately sent to the laboratory of Nanchang Center for Disease Control and Prevention at 4 °C after collection, while still in the storage medium. According to the manufacturer’s instructions, viral RNA was extracted from biological samples with QIAamp Viral RNA Mini Kit (Qiagen, Germany), Real-time RT-PCR were used for influenza typing and subtyping. The samples were identified as containing influenza A on the basis of the M gene, but could not be classified into subtypes. Specific real-time RT-PCR assays for avian influenza H5, H7, and H9 were done to verify the viral subtypes from nucleic acids positive to influenza A virus using commercial real-time PCR kit (Lifeliver, Shanghai).

### Statistical analysis

Use SPSS20.0 statistical software to sort out and analyze the monitoring data. The count data are expressed in frequency and percentage, and the positive detection rate of AIV among different groups is compared by the χ^2^ test, and P < 0.05 indicates that the difference is statistically significant.

## Results

### Total test results before and after vaccination

We collected 3 036 samples before vaccination and 2 160 samples after vaccination. The positive rates of AIV detection were 19.66% and 28.10%, respectively, and the positive rates after vaccination were significantly higher than those before vaccination (P < 0.001). H5, H7, H9 and untypable HA type infections were detected before vaccination, and the difference between them was statistically significant (P < 0.001), and the positive rate of H5 (0.37%) and H7 (0.00%) subtypes after vaccination was lower than before vaccination (P < 0.001). It is worth noting that the positive rate of H9 subtype (22.22%) and the positive rate of untypable HA type (5.51%) increased significantly after vaccination (P < 0.001) (Table 1).

**Table 1:** Detection of AIV in live poultry market in Nanchang City before and after vaccination

Period	N	No. Positive (%)	HA subtype (%)			
			H5	H7	H9	HA untyped
Before	3036	597 (19.66)	95 (3.13)	64 (2.11)	337 (11.10)	101 (3.33)
After	2160	607 (28.10)	8 (0.37)	0 (0.00)	480 (22.22)	119 (5.51)
Total	5196	1204 (23.17)	103 (1.98)	64 (1.23)	817 (15.72)	220 (4.23)
χ^2^		50.474	49.435	46.101	117.815	14.826
P		0.000	0.000	0.000	0.000	0.000

N, the total number of samples; No. Positive (%), number of positive samples of influenza A virus (positive rate of influenza A virus); HA subtype (%), number of positive samples for H5/H7/H9/ HA untyped (positive rate of H5/H7/H9/ HA untyped)

### Test results in different months before and after vaccination

**Fig. 1:**
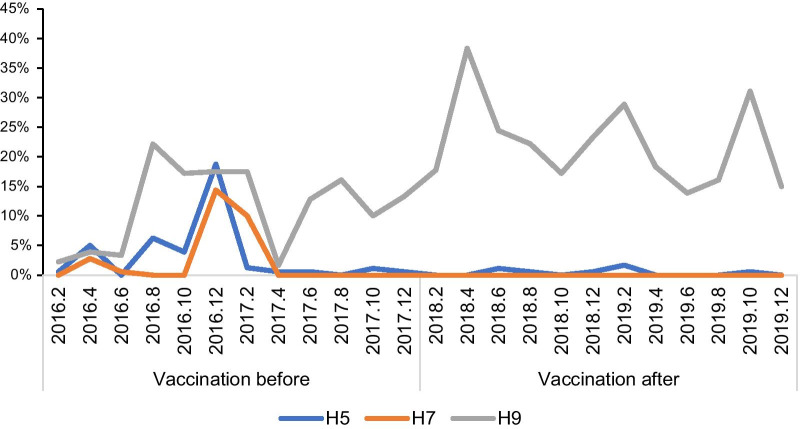
Changes of Avian influenza virus (AIV) in different months before and after vaccination. Values are expressed as a positive rate (%), positive rate = number of positive samples: total number of samples

Before vaccination, the positive rate of H5 and H7 subtype viruses peaked in December 2016 (18.75%) (14.38%), and the positive rate of H9 subtype viruses peaked in August 2016 (22.16%). At the beginning of 2017, the highly pathogenic avian influenza subtype H7N9 broke out. The Nanchang Municipal Government controlled the influenza virus outbreak by slaughtering sick poultry, cleaning the live poultry market, and closing the market. From February to April 2017, the H5, H7 and H9 subtypes all declined sharply. However, due to the previous measures such as closing the live poultry market and killing infected poultry, the positive rate of H5 and H7 subtypes has remained at a low value since then and the H9 subtype has been showing an upward trend overall. At the end of 2017, because live poultry were generally vaccinated with H5 + H7 avian influenza vaccine, the highly pathogenic AIV H5 and H7 subtypes did not rebound, and the positive rate remained low. However, the positive rate of H9 subtype was higher than that before vaccination, showing a rapid growth trend from December 2017 to April 2018, reaching the peak in April 2018 (38.33%), February 2019 (28.89%) and October 2019 (31.11%) respectively, and there will be a trough in summer (Fig. 1).

### Test results of different types of samples

**Fig. 2:**
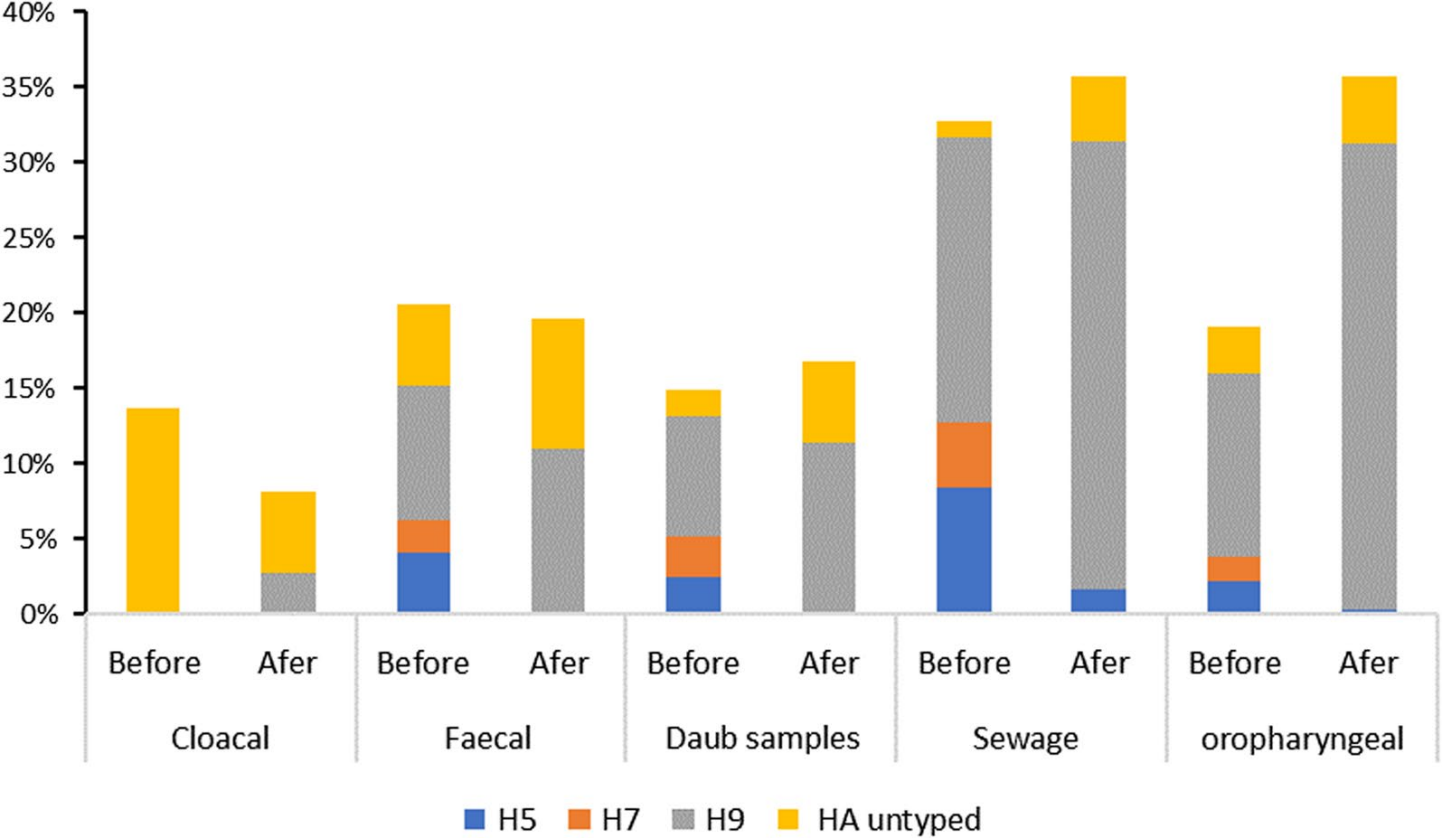
Changes in the positive rate (%) of AIV subtypes in different samples in Nanchang City before and after vaccination. Values are expressed as a positive rate (%), positive rate = number of positive samples: total number of samples. Before, vaccination before; After, vaccination after

Before and after vaccination, we collected a total of 2686 poultry samples (throat swabs, poultry cloaca samples) and 2510 environmental samples (feces of poultry, daub samples of cage surface, and poultry cleaning sewage). Among all samples, sewage samples have the highest positive rate of influenza A virus (33.90%) (P < 0.001). The changes in the positive rate of each subtype of environmental samples and poultry samples before and after vaccination were similar. After vaccination, the positive rates of H5 (0.36%) (0.38%) and H7 (0.00%) (0.00%) subtypes in poultry and environmental samples decreased (P < 0.001), while the positive rate of H9 (29.87%) (14.31%) subtype increased (P < 0.01). However, the positive rate of untypable HA type (6.59%) only increased significantly in environmental samples (P < 0.001), and there was no significant difference in poultry samples (Table 2). Further analysis showed that the positive rates of H5 and H7 subtypes decreased significantly in all environmental samples after vaccination (P < 0.01), and the positive rates of untypable HA type increased significantly in all environmental samples (P < 0.05). Since vaccination, the positive rate of H9 subtype of all samples has increased to varying degrees, but the positive rates of H9 subtypes (29.73%) only increased significantly in sewage samples (P < 0.01). After vaccination, the positive rate of H5 (0.38%), H7 (0.00%) and H9 (30.82%) subtypes was only significantly different in poultry throat swab samples (P < 0.001), but the positive rate of untypable HA type was not significantly different in poultry throat swabs and cloaca samples (Fig. 2).

**Table 2:** AIV detection results from poultry and environmental samples before and after vaccination

Period	N	No. positive (%)	HA subtype (%)			
			H5	H7	H9	HA untyped
**Poultry samples**						
Vaccination before	1588	299 (18.83)	33 (2.08)	24 (1.51)	185 (11.65)	57 (3.59)
Vaccination after	1098	381 (34.7)	4 (0.36)	0 (0)	328 (29.87)	49 (4.46)
**Environmental samples**						
Vaccination before	1448	298 (18.77)	62 (4.28)	40 (2.76)	152 (10.5)	44 (3.04)
Vaccination after	1062	226 (21.28)	4 (0.38)	0 (0)	152 (14.31)	70 (6.59)
Total	5196	1204 (23.17)	103 (1.98)	64 (1.23)	817 (15.72)	220 (4.23)

N, the total number of samples; No. Positive (%), number of positive samples of influenza A virus (positive rate of influenza A virus); HA subtype (%), number of positive samples for H5/H7/H9/ HA untyped (positive rate of H5/H7/H9/ HA untyped)

### Test results of different live poultry markets

A total of 2706 samples were collected from the wholesale market of live poultry and 2490 samples from the retail market of live poultry before and after vaccination. The positive rate of avian influenza A virus detected in the wholesale market of live poultry (21.73%) was lower than that in the retail market of live poultry (24.74%) (P < 0.05). In the two types of markets, the difference in the positive rate of the H5 subtype was not statistically significant, and the difference in the positive rate of the other subtypes was statistically significant. After vaccination, the positive rates of H5 (0.09%) (0.65%) and H7 (0.00%) (0.00%) subtypes in the wholesale and retail markets of live poultry decreased significantly (P < 0.001); while the positive rates of H9 subtypes (21.57%) (22.87%) and untypable HA type (7.13%) (3.89%) increased significantly after vaccination (P < 0.05), different types of live poultry markets have statistically significant differences in the positive rates of each subtype before and after vaccination (P < 0.05) (Table 3).

**Table 3:** AIV test results in wholesale and retail markets before and after vaccination [positive rate (%)]

Period	N	No. positive (%)	HA Subtype (%)			
			H5	H7	H9	HA untyped
**Wholesale market**						
Vaccination before	1626	277 (17.04)	48 (2.95)	20 (1.23)	142 (8.73)	67 (4.12)
Vaccination after	1080	311 (28.8)	1 (0.09)	0 (0)	233 (21.57)	77 (7.13)
**Retail market**						
Vaccination before	1410	320 (22.7)	47 (3.33)	44 (3.12)	195 (13.83)	34 (2.41)
Vaccination after	1080	296 (27.41)	7 (0.65)	0 (0)	247 (22.87)	42 (3.89)
Total	5196	1204 (23.17)	103 (1.98)	64 (1.23)	817 (15.72)	220 (4.23)

N, the total number of samples; No. Positive (%), number of positive samples of influenza A virus (positive rate of influenza A virus); HA subtype (%), number of positive samples for H5/H7/H9/ HA untyped (positive rate of H5/H7/H9/ HA untyped)

## Discussions

Control the influenza A (H7N9) virus that causes catastrophic death of poultry and large numbers of human infections. In addition to slaughtering poultry infected with the highly pathogenic H7N9 virus, since September 2017, reassortant AIV (H5 + H7) inactivated vaccines have also been vaccinated throughout China as one of the prevention and control measures [10]. Live poultry in Nanchang City have also fully implemented immunization in accordance with the plan.

Through active monitoring for 4 consecutive years before and after vaccination, we found that after H5 + H7 influenza vaccination, the positive rate of H5 and H7 subtypes showed a downward trend, and even dropped to 0. Some studies have similar results. [8, 10]. This shows that measures taken by the government's health and related departments, such as vaccination of avian influenza, catching and killing infected poultry, and cleaning the live poultry market, can effectively curb the growth of highly pathogenic AIV subtypes. At the beginning of vaccination, H9 subtype may gain a competitive advantage, increase rapidly, and then have a trough in summer, which is related to the characteristics of H9 subtype [17]. But this rise deserves our vigilance. Unlike H5 and H7 subtypes, which cause high mortality and high pathogenicity, low pathogenicity H9 (H9N2) subtypes generally do not cause significant clinical symptoms or death in infected poultry [18]. This will bring challenges to the identification and control of infected poultry, and promote the rapid spread of H9 subtype virus in the LPM. Although the H9 subtype is classified as a low pathogenic AIV, some studies have found that some H9N2 viruses are highly lethal to mice and can spread systemically, similar to the highly pathogenic avian influenza virus (HPAIV) [19, 20]. It is well known that the low pathogenic H9 (H9N2) subtype provides internal genes for H5N1, H7N9 or H10N8 viruses that have caused fatal human infections since 2013 [21–23]. For example, a new type of H3N6 virus was isolated from migratory birds in Poyang Lake, Nanchang City, and it was discovered that the H9N2 virus contributed the PB1 gene to the new type of virus [24]. And the H9 (H9N2) subtype can also spread across species [25, 26], and has been confirmed to infect humans [17, 27]. However, patients usually show only mild and typical human influenza like diseases, which is easy to be ignored [28]. So in fact, the number of people infected with H9N2 virus is far greater than the number of confirmed cases. After vaccination, the H5 and H7 subtypes are suppressed, and the H9 subtype gains a competitive advantage, leading to an increase in the positive rate of the H9 subtype. Therefore, this may be the reason why the positive rate of type A avian influenza virus has not decreased after vaccination. If the H9 subtype continues to maintain a high positive rate, it will pose a huge potential threat to public health. However, in order to reduce this threat, we must take certain measures to reduce the high positive rate of the H9 subtype virus.

The determination of the viral load and subtypes of different poultry oropharyngeal swabs and cloacal swabs may help to understand the impact of viruses carried by poultry on the number of viruses released in the environment [29]. Our results show that the positive rates of poultry samples and environmental samples are similar. Therefore, the high positive rate of some subtypes in poultry samples may be the main contributing factor of subtypes detected in the environment, while environmental pollution in turn may pose a great potential threat to healthy poultry and other species, and may cause mixed infection of infected live poultry. This may result in the continued spread of the virus in the LPM, thus increasing the risk of human infection with AIV. The H9 subtype positive rate of environmental sewage samples changed the most after vaccination. Nanchang City is located in southern China, the air is already humid, and the humid environment will affect the survival time of the AIV [30]. During the slaughter process of live poultry, aerosols containing virus particles may be generated and spread in a narrow, poorly ventilated space [31], which can easily cause environmental pollution in the live poultry market. If the contaminated sewage is not disinfected in time, it is very easy to produce the accumulation effect of AIV. Studies have shown that [30] subtypes in the air have a good correlation with those in the environment (water, feces and smear samples), so the high concentration of AIV accumulated in the environment will pollute the surrounding air, thus indirectly increasing the possibility of poultry spreading to humans. It has been reported that [32] some infected patients, without direct or indirect contact with poultry, just went to live poultry market but were infected with AIV. And in the poultry market, people have more opportunities to be exposed to the environment. Therefore, compared to poultry infected with AIV, an environment with a high viral load is more likely to pose a threat to poultry practitioners and customers. Laboratory confirmed cases of human infection with the H9 (H9N2) subtype virus have been reported sporadically from the WHO, and the incidence has been significantly higher in the past few years. In China, only in the first half of 2021, there were 9 human cases of H9N2 infection, while only one case of H5N6 occurred, and H7N9 was zero infection [33]. Therefore, the high detection rate of the H9 subtype should be valued by us, and the monitoring, prevention and control of AIVs at this stage cannot be slackened.

Our test results show that the positive rate of AIV in the live poultry wholesale market (21.76%) is lower than the AIV positive rate in the retail market (24.62%). This confirms that the detection rate of AIV mentioned in some studies [27] has increased with the increase of the live poultry supply chain, and a large number of AIVses have been spread and accumulated for a long time at the end of the live poultry transaction. Due to the aggregation of different types of live poultry in the retail market, dense stalls, poor air circulation, and untimely treatment of ground pollutants and sewage, poultry raising, slaughtering and sales are not divided into different areas. This may have promoted the spread of AIV in the live poultry retail market [34], resulting in a higher AIV positive rate in the retail market. In the live poultry wholesale market, different types of live poultry are placed separately, with sufficient space, less dirt and other reasons, which may slow the spread of the virus at a certain level. We understand that poultry workers in the retail market also have higher AIV serum antibodies than poultry workers in other environments [27]. This suggests that more attention should be paid to the daily management of the live poultry retail market, and LPM should be sealed and disinfected regularly to prevent the spread of AIV to live poultry workers and the general population. After vaccination, both the live poultry retail market and the wholesale market, the positive rates of H9 subtypes and untypable HA type have increased significantly. This shows that even if vaccination measures are taken, cleaning and disinfection measures at all levels still need to be improved to reduce virus amplification. And as long as the live poultry trade continues, the role of the live poultry market as a reservoir of AIV and gene pool will not be fundamentally changed. Although the H9 subtype AIV is classified as a low pathogenic AIV, it is widely distributed in the live poultry market, which will pose a continuous challenge to poultry and humans [33]. Studies have shown that after the live poultry market is closed, the number of H9 (H9N2) viruses detected has decreased [30]. The best way to reduce exposure and infection is to close the live poultry market and implement centralized slaughtering and marketing. At the same time, it can also provide additional protection for the poultry supply chain from farm to table and reduce related economic losses.

## Conclusions

For H9 (H9N2) subtype, China's long-term vaccination program still caused huge economic losses to the chicken industry [17]. Therefore, in response to the rapidly increasing H9 subtype AIV, we should focus on other more sustainable and effective interventions besides vaccines to control the spread of AIV [30, 33, 35, 36]. For example, daily washing and cleaning of slaughtering utensils, weekly disinfection of the environment, separate placement of different kinds of live poultry such as waterfowl and land poultry, and regular closure of live poultry market every month can simultaneously reduce the risk of AIV pollution from the source, which is worthy of priority. Therefore, it is more important to establish good biosafety management and take all practical measures to control the source of infection and prevent virus strains from invading poultry.

## Data Availability

The datasets used and/or analyzed during the current study are available from the corresponding author on reasonable request.

## Abbreviations

AIV: Avian influenza virus
LPM: Live poultry markets
